# Detection and quantification of *Spirocerca lupi* by HRM qPCR in fecal samples from dogs with spirocercosis

**DOI:** 10.1186/s13071-017-2374-3

**Published:** 2017-09-19

**Authors:** Alicia Rojas, Gilad Segev, Alex Markovics, Itamar Aroch, Gad Baneth

**Affiliations:** 1 Koret School of Veterinary Medicine, The Hebrew University of Jerusalem, P.O. Box 12, 7610001 Rehovot, Israel; 2 Kimron Veterinary Institute, 50250 Bet Dagan, Israel

**Keywords:** *Spirocerca lupi*, Spirocercosis, PCR, ITS1, 18S, *Cytb*, Canine, Coproscopy

## Abstract

**Background:**

*Spirocerca lupi*, the dog oesophageal nematode, causes a potentially fatal disease in domestic dogs, and is currently clinically diagnosed by coproscopy and oesophagoscopy. To date, a single molecular method, a semi-nested PCR, targeting the *cox*1 gene, has been developed to aid in the diagnosis of spirocercosis. The present study describes three novel high-resolution melt (HRM) quantitative PCR (qPCR) assays targeting fragments of the ITS1, 18S and *cytb* loci of *S. lupi*. The performance of these molecular assays in feces was compared to fecal flotation and to the previously described *cox*1 gene semi-nested PCR in 18 fecal samples from dogs with clinical oesophageal spirocercosis diagnosed by oesophagoscopy.

**Results:**

The HRM qPCR for ITS1 and 18S were both able to detect 0.2 *S. lupi* eggs per gram (epg), while the HRM qPCR for the *cytb* and the semi-nested PCR for the *cox*1 detected 6 epg and 526 epg, respectively. *Spirocerca lupi* was detected in 61.1%, 44.4%, 27.8%, 11.1% and 5.6% of the fecal samples of dogs diagnosed with spirocercosis by using the ITS1 and 18S HRM qPCR assays, fecal flotation, *cytb* HRM qPCR and *cox*1 semi-nested PCR, respectively. All dogs positive by fecal flotation were also positive by ITS1 and 18S HRM qPCRs. Quantification of *S. lupi* eggs was successfully achieved in the HRM qPCRs and compared to the fecal flotation with no significant difference in the calculated concentrations between the HRM qPCRs that detected the 18S and ITS1 loci and the fecal flotation. The HRM qPCR for the 18S cross-amplified DNA from *Toxocara canis* and *Toxascaris leonina.* In contrast, the HRM qPCR for ITS1 did not cross-amplify DNA from other canine gastrointestinal parasites.

**Conclusions:**

This study presents two new molecular assays with significantly increased sensitivity for confirming and quantifying fecal *S. lupi* eggs. Of these, the HRM qPCR for ITS1 showed the best performance in terms of the limit of detection and absence of cross-amplification with other parasites. These assays will be useful in detecting infection and for follow-up during therapy.

**Electronic supplementary material:**

The online version of this article (10.1186/s13071-017-2374-3) contains supplementary material, which is available to authorized users.

## Background

*Spirocerca lupi*, the dog oesophageal nematode, is the etiological agent of spirocercosis, a chronic, potentially life-threatening disease of carnivores, predominantly of canids, including the domestic dog [1]. It has an indirect life-cycle which involves dung beetles as intermediate hosts and canids as definitive hosts. In the dog, *S. lupi* adults typically localize within oesophageal nodules and shed eggs to the host’s gastrointestinal tract and in the feces [1]. Spirocercosis has been reported in the tropical and subtropical areas of Europe, Africa, Asia and the Americas, with incidences ranging from 5.4% in South Africa [2] to 25% in Iran [3]. The annual incidence in Israel ranges from 0.7 to 8% [4].

Spirocercosis in dogs is suspected based on the history, clinical signs and imaging findings (i.e. thoracic survey radiography and computed tomography), and diagnosis is currently confirmed by coproscopy, using several fecal flotation methods and oesophagoscopy. However, due to the chronicity of this disease, dogs are usually diagnosed in advanced disease stages, which are associated with severe detectable clinical signs [5]. Moreover, clinical signs of spirocercosis are variable, depending on the migratory routes of the worm, occurrence of the transformation of benign to malignant nodules and the parasite intensity. The serological cross-reactivity between *S. lupi* and *Dirofilaria immitis* circulating antigen [6] adds to the challenges in the correct diagnosis of spirocercosis [1].

Three laboratory-based diagnostic tools have been described to date for detecting *S. lupi* infection. These methods include microscopic examination of feces for the identification of *S. lupi* eggs using flotation methods, such as a technique based on a concentrated sugar solution [7], an immunofluorescence antibody test for detecting *S. lupi* antigens, which is commercially unavailable, and its utility in clinical practice is unknown [8], and a semi-nested PCR, targeting a 400 bp fragment of the mitochondrial *cox*1 gene [5]. These methods, however, have limitations. The fecal flotation method requires expertise in distinguishing *S. lupi* eggs from eggs of other helminths and may produce false negatives due to intermittent egg shedding in the feces [1], while the semi-nested PCR is susceptible to contamination [9, 10]. Thus, sensitive and specific diagnostic tools are needed for early detection of *S. lupi* in infected dogs.

Quantitative real-time PCR (qPCR) assays provide a very sensitive tool for diagnosing and quantifying parasitic infections [11]. In addition, qPCR, followed by high-resolution melt analysis (HRM), is a useful tool for the specific identification of DNA amplicons based on their melting temperatures, thereby facilitating rapid diagnosis [11]. The present study explored the use of HRM qPCR assays for the specific diagnosis and quantification of *S. lupi* in dogs with naturally occurring spirocercosis. Accordingly, three different HRM qPCRs, targeting fragments of the 18S ribosomal RNA gene, the internal transcribed spacer 1 (ITS1) loci and cytochrome *b* gene (*cytb*) of *S. lupi,* were developed. In addition, their performance was compared with the currently available diagnostic methods, namely coproscopy by fecal flotation and the *cox*1 semi-nested PCR. Lastly, the potential cross-amplification of other canine parasitic nematode DNA was evaluated for all the HRM qPCR assays.

## Methods

### *Spirocerca lupi* infected dogs and fecal flotation

Fecal samples were collected from 18 dogs (11 males and 7 females), with ages ranging between 1 and 13 years, untreated previously with anthelminthic drugs, and diagnosed with clinical spirocercosis. Oesophageal spirocercosis was suspected based on compatible history and clinical signs and confirmed by characteristic oesophagoscopy findings in all dogs. The latter included identifying oesophageal nodules with a smooth appearance, containing a characteristic nipple-like protuberance [1]. Dogs with neoplastic transformation were excluded [1]. Then, the dogs were treated, and the diagnosis was further confirmed by repeated oesophagoscopy, at weeks 16 to 20 after the initial oesophagoscopy, during treatment to monitor the response to therapy. Fecal samples were examined by a double centrifugation fecal flotation, using sugar solution with a specific gravity of 1.27, as previously described [7], and the number of eggs per gram (epg) of feces was determined in all the positive fecal samples. Approximately 200 mg of each fecal sample were placed in a microcentrifuge tube, and stored at -80 °C pending further analyses.

### Collection of eggs from adult *S. lupi*-female worms

*Spirocerca lupi* eggs were obtained from female adults maintained in an in vitro culture, as previously described [12]. The growth medium was collected after 24 h of incubation, and the presence of shed eggs contained in the solution was confirmed by microscopic examination. The eggs were maintained at 4 °C pending further analyses. The concentration of the egg suspension was adjusted to 14,200 epg by dilution with the growth medium solution.

### DNA extraction of *S. lupi* samples

DNA was extracted from 200 μl of the in vitro obtained *S. lupi-*egg suspensions and from 200 mg of the dogs’ fecal samples using the QIAamp Fast DNA Stool Mini Kit (Qiagen, Hilden, Germany), according to the manufacturer’s instructions, with the following modifications. Briefly, the weight of each fecal sample used for the extraction was recorded to estimate the egg concentration by the molecular methods. Suspension with the InhibitEX buffer (Qiagen) was heated at 95 °C for 15 min with light mixing by vortex every 5 min. After incubation, five sterile glass beads were added to each sample and mixed by vortex for 10 min. The suspension was then centrifuged at 14,000× *rpm* for 1 min, and 200 μl of the supernatant was incubated with AL buffer (Qiagen) and proteinase K (Qiagen) for 20 min, at 70 °C, after which, all steps as recommended by the manufacturer were followed. DNA purity and concentration was verified using a NanoDrop spectrophotometer (Thermo-Fisher Scientific, Massachusetts, USA).

### *Spirocerca lupi* standard curve

DNA from the suspension with 14,200 epg of *S. lupi* was serially diluted 3-fold, to a final volume of 100 μl in PCR-grade water (Biological Industries, Kibbutz Beit-Haemek, Israel). In total, 12 *S. lupi-*standard concentrations, ranging from 14,200 to 0.07 epg, were prepared for the qPCR analyses. The egg standards were run in triplicates to build a standard curve, and estimate the detection limit, slope, intercept, linear regression coefficient and efficiency for each of the HRM qPCRs.

### HRM qPCR assays for the detection of *S. lupi* DNA

#### HRM qPCR for the ITS1 spacer

The *S. lupi* ITS1 spacer was characterized from four adult *S. lupi* specimens using primers rDNA2 [13] and rDNA1.58S [14], following a previously described protocol [14]. Then, the amplified sequence was inserted into pCR2-TOPO vectors (Thermo Fisher Scientific Inc.) and cloned. Specific primers were designed using Primer-BLAST [15], to amplify a 135 bp fragment within the ITS1 of *S. lupi.* The HRM qPCR assay used primers SlITS-F (5′-AAA CGG TGT CCC ATG TTG G-3′) and SlITS-R (5′-GCA GCA CAA TAG CTT GAC GC-3′) at a final concentration of 500 nM. Each reaction consisted of 1 μl of each primer, 0.6 μl of SYTO-9 (Invitrogen, California, USA), 4.4 μl of PCR grade water (Biological Industries Inc.), 10 μl of Maxima HotStart Master Mix® (Thermo Fisher Scientific Inc.) and 3 μl of DNA. The amplification reaction consisted of an initial hold at 95 °C for 4 min, followed by 45 cycles of 95 °C for 5 s, 60 °C for 15 s and 72 °C for 10 s. A melt curve from 80 °C to 95 °C was constructed with increments of 1 °C/s, followed by a hybridization step from 90 °C to 50 °C. An HRM curve was constructed from 70 °C to 85 °C, with 0.1 °C/s increments.

#### HRM qPCR for the 18S locus

Primers for a fragment of the 18S locus of *S. lupi* available in GenBank (HQ674750.1) were designed using Primer-BLAST [15]. Accordingly, primers Sl18S-F (5′-AAG CTC CGA CTT TTG GAC GA-3′) and Sl18S-R (5′-GTC ACT ACC TCC TCA TGC CG-3′) were constructed to amplify a 270 bp fragment of the 18S gene and used at a final concentration of 500 nM. The reactions consisted of 1 μl of each primer, 0.6 μl of SYTO-9 (Invitrogen), 4.4 μl of PCR grade water (Biological Industries Inc.), 10 μl of Maxima HotStart Master Mix® (Thermo Fisher Scientific Inc.) and 3 μl of DNA. The amplification reaction consisted of an initial hold of 95 °C for 4 min, followed by 50 cycles of 95 °C for 5 s, 59 °C for 15 s and 72 °C for 10 s. A melt curve from 80 °C to 95 °C was constructed with 1 °C/s increments, followed by a hybridization step from 90 °C to 50 °C. An HRM was constructed from 80 °C to 90 °C with 0.1 °C/s increments.

#### HRM qPCR for the *cytb* locus

A sequence of the *cytb* gene from GenBank (KC305876.1) [16] was used for the development of novel primers for this locus. Hence, primers Slcytb-F (5′-ACT GCG GGG GAG TCT TTC T-3′) and Slcytb-R (5′-AGT AAT AAC AAC CGC CGC CC-3′) were designed to amplify a 260 bp fragment of the *cytb* gene. Primers were used at a final concentration of 250 nM. Each reaction consisted of 0.5 μl of each primer, 0.6 μl of SYTO-9 (Invitrogen), 5.4 μl of PCR grade water (Biological Industries Inc.), 10 μl of Maxima HotStart Master Mix® (Thermo Fisher Scientific Inc.) and 3 μl of DNA. The program consisted of an initial hold at 95 °C for 4 min and 45 cycles of 95 °C for 5 s, 57 °C for 15 s and 72 °C for 5 s. A melt curve from 75 °C to 90 °C was constructed, with 1 °C/s increments, followed by a hybridization step. The HRM curve was constructed from 75 °C to 90 °C with 0.1 °C/s increments.

All HRM qPCRs were run in the Rotor Gene 6000 Cycler (Qiagen, Hilden, Germany). Each reaction was run with positive, negative and non-template controls (NTC). Reaction controls included *S. lupi* adult-DNA (positive control), DNA from a *S. lupi*-negative dog fecal sample (negative control, determined by fecal flotation in three consecutive samples), a point of the standard curve by triplicate and NTC with PCR-grade water.

### Semi-nested PCR for detection of the *S. lupi cox*1 gene

The semi-nested PCR for the amplification of a fragment of the *cox*1 gene of *S. lupi* was performed as described previously [5], with the following modifications to improve the detection of *S. lupi* DNA, based on preliminary tests. In the first round of amplification, primers NTF and NTR were employed at a final concentration of 200 nM instead of 2 pmol, while MgCl_2_ was used at a final concentration of 3 mM, and finally, 3 μl of DNA standard, were added to each reaction in PCR-ready Syntezza® tubes (Syntezza Bioscience Ltd., Jerusalem, Israel). In the nested PCR reaction, the SlInt and NTR primers were used at a final concentration of 200 nM, instead of 2 pmol, while MgCl_2_ was used at a final concentration of 3 mM. DNA from the first PCR reaction was diluted 40-fold in PCR-grade water, and 5 μl of this dilution was employed as a template. The PCR cycling protocol consisted of an initial hold at 95 °C for 5 min, followed by 40 cycles of 94 °C for 45 s, 54 °C or 60 °C for 1 min in the primary and secondary reaction, respectively, and 72 °C for 1 min, and a final elongation at 72 °C for 5 min. The fecal samples from the dogs with spirocercosis were run together with *S. lupi* adult-DNA, a *S. lupi*-negative dog fecal control and an NTC with PCR-grade water. Additionally, DNA standards of *S. lupi* were run in triplicate to estimate the assay detection limit.

### Experimental controls

DNA from the following gastrointestinal parasites was used as controls to evaluate the possible cross-amplification of the *S. lupi* HRM qPCR assays: *Ancylostoma caninum*, *Calodium hepaticum*, *Strongyloides stercoralis*, *Toxocara canis*, *Toxascaris leonina*, *Taenia solium*, *Dyphillobothrium latum*, *Dipylidium caninum*, *Spirometra mansoni*, *Echinococcus granulosus*, *Echinococcus multilocularis*, *Giardia duodenalis*, *Neospora caninum* and *Cystoisospora canis.* The life stages, sources and DNA-extraction methods of each of these parasites are indicated in Additional file 1: Table S1. Additionally, an experimental control mimicking severe toxocariasis infection with *T. canis* [17] was prepared. Briefly, a concentrated egg suspension of *T. canis* was obtained from the uterus of four adult female *T. canis* worms. The eggs were counted in triplicate, and their concentration adjusted to 93,000 epg.

### Sequence analysis

Positive DNA amplicons were purified (Exo-SAP New England Bio-Labs Inc., Ipswich, USA) and sequenced using the Big-Dye Terminator cycle sequencing chemistry from Applied Biosystems ABI3700 DNA Analyzer and the ABI’s Data Collection and Sequence Analysis software (ABI Carlsbad, USA). Samples were considered positive for *S. lupi* when the obtained amplicon had at least 97% sequence identity, and the closest match was to *S. lupi* sequences available in GenBank using the BLAST 2.2.28 program.

### Data analysis

Each positive sample was run in triplicate, to calculate the geometric mean of *S. lupi* epg and standard deviation. Fecal samples were considered positive when all three replicates were amplified. The HRM qPCRs C_q_ values of each positive sample were used to estimate the *S. lupi* epg, as in the following formula:

$$ epg={10}^{\frac{Cq-b}{m}}\times Cf $$

C_q_ stands for quantification cycle, b for y-intercept, m for the slope obtained in the standard curve, and Cf is a correction factor, calculated for each fecal sample. The average C_q_ value of each positive sample was interpolated in the standard curve equation to obtain the epg (first factor of the equation). The variability in the mass of fecal sample employed for DNA extraction was normalized by dividing 0.2 by the exact weight of the extracted fecal sample (in g).

### Statistical analysis

Cohen’s kappa coefficient was calculated to determine the agreement in the detection of spirocercosis cases between the four molecular assays and the fecal flotation. Κ-values were interpreted according to Landis & Koch [18]. The Wilcoxon signed rank test was used to determine the differences in the epg concentration in the fecal samples of the dogs with spirocercosis obtained by fecal flotation and the estimated values from the HRM qPCRs. This same test was used for comparing the epg obtained in the HRM qPCRs for the ITS1 and 18S. Spearman’s ranks correlation coefficient was calculated to determine the correlation between the epg as quantified by the ITS1 and 18S HRM qPCRs*.* Lastly, Bonferroni’s correction was applied for multiple comparisons. Significance was considered when *P <* 0.017. Statistical analyses were performed using the SPSS 22.0 software package (IBM Corp., USA).

## Results

### HRM real time qPCR assays performance

The standard curve for the ITS1, 18S and *cytb* HRM qPCR assays showed efficiencies of 90%, 103% and 134%, respectively. The y-intercept, slope and linear correlation (R^2^) are shown in Table 1.

**Table 1:** Characteristics of the HRM qPCR assay performances

Parameter	PCR assay		
	HRM qPCR for ITS1	HRM qPCR for 18S	HRM qPCR for *cytb*
Efficiency (%)	90	103	134
Slope	-3.61	-3.23	-2.70
y-intercept	35.15	34.88	40.12
R^2^ value	0.9976	0.9962	0.9896

The HRM qPCRs for the ITS1 and 18S loci showed detection limits of 0.2 epg. The HRM qPCR for the *cytb* detected down to 6 epg. In contrast, the detection limit of the *cox*1 semi-nested PCR with the employed modifications was 526 epg (Additional file 2: Figure S1). The designed primers for ITS1 did not cross-amplify any DNA from the other examined common gastrointestinal dog parasites. The HRM qPCR for the 18S gene amplified DNA from *T. canis* and *T. leonina* after 30 cycles. *Dipylidium caninum* DNA was amplified in the HRM qPCR for *cytb* but only after cycle 30.

### Detection of spirocercosis

The molecular methods targeting ITS1 and 18S of *S. lupi* detected higher numbers of positive fecal samples than the currently available diagnostic methods (i.e. fecal flotation and semi-nested *cox*1 PCR). The HRM qPCR for ITS1 and 18S were positive in 61.1% (11/18; 95% CI: 35.7–82.7%) and 44.4% (8/18; 95% CI: 21.5–69.2%) of the fecal samples from the dogs with spirocercosis, respectively. The fecal flotation assay identified *S. lupi* eggs in 27.8% of the dogs (5/18; 95% CI: 9.7–53.5%), while the HRM qPCR for *cytb* and the semi-nested *cox*1 PCR identified only two and one samples as positive, respectively.

Agreement for the detection of positive and negative samples for *S. lupi* was observed between the methods. There were fair (κ = 0.39) and good (κ = 0.65) agreements between the HRM qPCR for ITS1 and the fecal flotation, and between the HRM qPCR for 18S and the fecal flotation, respectively. The HRM qPCRs for ITS1 and 18S were positive in all the fecal samples found to have *S. lupi* eggs by the fecal flotation (Table 2). Furthermore, 6 and 3 samples from dogs with spirocercosis detected positive by the HRM qPCR for ITS1 and 18S, respectively, were negative by the fecal flotation. The fecal flotation results had a moderate agreement with the *cytb* HRM qPCR (κ = 0.50) and fair agreement with the semi-nested PCR for the *cox*1 (κ = 0.26). The semi-nested *cox*1 PCR and the HRM *cytb* qPCR assay were positive in 10% and 20%, respectively, of the samples detected as positive by the fecal flotation. When comparing the HRM qPCRs for ITS1 and 18S, their agreement was good (κ = 0.67). There was none to slight agreement when comparing the results of ITS1 HRM qPCR and *cytb* HRM qPCR (κ = 0.15) and of the semi-nested *cox*1 PCR (κ = 0.16), as well as between the 18S HRM qPCR and the semi-nested *cox*1 PCR (κ = 0.14).

**Table 2:** Detection of *S. lupi* eggs in fecal samples from dogs by five different assays

Fecal sample code number	HRM qPCR for ITS1 (estimated epg)	HRM qPCR for 18S (estimated epg)	HRM qPCR for *cytb* (estimated epg)	Semi-nested PCR for *cox*1	Fecal flotation (estimated epg)
A-01F	+ (13.1 ± 9.0)	+ (2.2 ± 0.1)	–	–	+ (120)
C-01F	–	–	–	–	–
D-01F	+ (106.8 ± 45.5)	+ (28.5 ± 8.2)	+ (5.7 ± 1.8)	+	+ (10)
E-01F	+ (0.7 ± 0.1)	+ (0.03 ± 0.001)	–	–	+ (12)
F-01F	–	–	–	–	–
G-01F	–	–	–	–	–
I-01F	+ (7.8 ± 1.0)	+ (0.3 ± 0.04)	–	–	–
J-01F	+ (0.7 ± 0.1)	–	–	–	–
K-01F	+ (0.5 ± 0.1)	–	–	–	–
L-01F	–	–	–	–	–
M-01F	+ (0.7 ± 0.1)	–	–	–	–
N-01F	+ (2.3 ± 0.5)	+ (0.3 ± 0.1)	–	–	+ (1)
O-01F	+ (41.4 ± 9.8)	+ (17.0 ± 3.1)	+ (0.1 ± 0.06)	–	+ (160)
P-01F	–	–	–	–	–
Q-01F	–	–	–	–	–
S-01F	–	–	–	–	–
T-01F	+ (8.6 ± 2.2)	+ (0.97 ± 1.1)	–	–	–
U-01F	+ (12.3 ± 1.5)	+ (4.4 ± 0.1)	–	–	–
Total	11	8	2	1	5

Repeated oesophagoscopies of the dogs included in this study indicated that the oesophageal nodules in all 18 dogs had resolved after 16-20 weeks of treatment, proving that spirocercosis was benign.

### Quantification of *S. lupi* eggs from fecal samples

Quantification of *S. lupi* eggs was successfully achieved by the HRM qPCRs for ITS1, 18S and *cytb* loci, and by the fecal flotation (Table 2). No significant difference in the calculated concentrations was found between the results when all these assays were compared (Wilcoxon signed rank test; *P* > 0.05 for all). However, when comparing the eggs quantified by the HRM qPCR for ITS1 and 18S, there was a significant difference (Wilcoxon signed rank test, *W* = -2.803, *P* = 0.005) with a significant positive correlation (Spearman’s *r*_*s*_ = 0.918; *P* < 0.0001; Additional file 3: Figure S2).

### Sequence analysis

All fecal samples found positive by the HRM qPCRs for 18S, and *cytb* loci were identified as containing *S. lupi* DNA by sequence comparison to publicly available sequences on the GenBank database. The accession number with the closest match and percentage of identity in the 18S HRM qPCR was HQ674750.1 (99%), and for the *cytb* qPCR and *cox*1 semi-nested PCR, it was KC305876.1 (97% and 96%, respectively). No *S. lupi* ITS1 sequence has been deposited in GenBank, and the closest match was *Cylicospirura petrowi* (KM434335.1, 96% identity with 47% coverage). Representative *S. lupi* ITS1, 18S, *cytB* and *cox*1 sequences obtained in this study were deposited in GenBank under accession numbers MF425539, MF79497, MF804403 and MF804404, respectively.

## Discussion

The current diagnosis of spirocercosis is challenging in clinical practice due to the variable nature of the clinical signs related to the different clinical presentations, parasite migration and development within the host, and the intermittent, unpredictable shedding of eggs in the feces [1]. Thus, to assess and improve the diagnosis of oesophageal spirocercosis, the present study explored the use of HRM qPCR assays to detect and quantify fecal *S. lupi* eggs. Accordingly, three novel HRM qPCRs were designed, and fecal samples from dogs confirmed with typical *S. lupi* oesophageal nodules were tested and compared with two currently available methods, namely, conventional coproscopy by the fecal flotation [7] and the *cox*1 semi-nested PCR [5]. The detection of fecal *S. lupi* DNA of dogs with oesophageal spirocercosis was successful and efficiently achieved in the HRM qPCRs targeting ITS1 and 18S.

The ribosomal (rDNA) and mitochondrial (mtDNA) DNA are among the most frequently used targets for PCR, due to their high copy number and their ability to display nucleotide sequence variability among species, which facilitates identification of parasite species [19]. The three molecular protocols described in this study, two associated with rDNA and one with mtDNA, successfully amplified *S. lupi* DNA. The HRM qPCR designed for ITS1 demonstrated the best potential for the diagnosis of *S. lupi* DNA and did not cross-amplify DNA of other gastrointestinal parasites, as compared to the 18S and *cytb* assays. The ITS1 and 18S assays exhibited the same detection limit (0.2 epg), in contrast to the *cytb* assay which showed a 30-fold higher minimal detection limit (6 epg). The diagnostic utility of the ITS1 and 18S assays might be explained by a high loci copy number or good sequence homology to the primers used and better reaction conditions. Nevertheless, the ITS1 assay detected the most spirocercosis cases in this study (Table 2). In the case of the *cytb* assay, several primer pairs and conditions were tested, but the sensitivity of this assay could not be further improved.

The currently available methods, fecal flotation and the semi-nested *cox*1 PCR, demonstrated a much lower performance than the molecular methods described in this study, with the exception of the *cytb* assay. The higher performance of the ITS1 and 18S assays in comparison to the fecal flotation likely results from the superior sensitivity of DNA-detection methods compared to microscopic methods, which require the detection of intact eggs. A similar scenario was observed in the detection of microfilariae DNA by PCR when compared with whole microfilariae microscopic identification in canine blood samples [20]. The HRM qPCRs developed for this study may be able to detect DNA from disrupted eggs, worm tissue parts or worm cellular DNA discharged into the host’s gastrointestinal lumen. Lastly, the molecular methods use DNA extracted from a fecal volume which is 15-fold smaller than that required for the fecal flotation. Nevertheless, the ITS1 and 18S HRM qPCR assays identified all the cases found positive by fecal flotation, as well as additional ones that were negative by fecal flotation but were positive by oesophagoscopy.

The semi-nested PCR had the highest detection limit in this study. To date, this was the only molecular assay described for detecting fecal *S. lupi* DNA [5]. However, nested-PCR protocols are usually more time consuming, requiring two reactions, and are prone to contamination, as reported in the diagnosis of viruses and bacteria of clinical importance [9, 10]. Therefore, the protocols designed herein demonstrated superior laboratory efficiency, requiring only a single reaction and being more effective in detecting spirocercosis than the nested PCR.

Oesophagoscopy is considered the most sensitive and specific diagnostic technique for the detection of oesophageal spirocercosis [21], but is limited by high cost, availability of specialized equipment, requirement for general anesthesia, potential complications (e.g. oesophageal bleeding or rupture) and the need for clinical expertise for the accurate identification of *S. lupi* nodules [1, 5]. Although in the present study, up to 61% of the spirocercosis cases were detected as positive by the ITS1 HRM qPCR, seven cases verified by oesophagoscopy were not detected as positive by any other methodology. This might be explained by the intermittent egg shedding into the feces, and low fecal egg numbers, which might have been absent in the small volume of fecal sample obtained for DNA extraction, or single-sex worm oesophageal infection, resulting in the absence of egg shedding [22]. Thus, we recommend analyzing a second fecal sample, obtained a few days after a negative result, since this can increase the positivity of detection in fecal samples, as previously suggested [21, 22].

The new HRM qPCRs might be helpful not only for detecting and quantifying fecal *S. lupi* but also for monitoring therapy and its effectiveness, as evident by the decrease and eventual absence of fecal *S. lupi* eggs and DNA in a previous study [23]. Previous studies in experimentally infected dogs have shown that fecal samples became negative for *S. lupi* eggs within 3 to 10 days after the first or second doramectin dose, given at a 2-week interval [23]. In naturally infected dogs, it has been shown that fecal samples remain positive for *S. lupi* eggs for up to 4 weeks after treatment with levamisole hydrochloride [24]. Therefore, the new molecular techniques, which have demonstrated the ability to detect very small amounts of fecal *S. lupi* DNA, equivalent to less than one epg, should be considerably more sensitive for detecting persistent infection than conventional coproscopy.

Interestingly, the HRM qPCR for ITS1 quantified a higher number of eggs than the assay for 18S (Table 2 and Additional file 3: Figure S2), although these loci are located in the same ribosomal operon, and should, therefore, be present in identical numbers of copies. This discrepancy might result from a higher annealing specificity of the primers to their target sequence in the case of the ITS1.

The HRM qPCR for ITS1 has amplified *S. lupi* DNA exclusively, whereas the 18S and *cytb* HRM qPCRs also amplified DNA from other canine gastrointestinal parasites. The cross-amplification of *T. canis* and *T. leonina* by the 18S HRM qPCR might be explained by a sequence similarity between the two nematodes, and because for *T. canis,* DNA from a very high egg concentration was employed, to simulate severe infection [25]. Since the ITS1 HRM qPCR was the only assay which did not cross-amplify other parasite DNA, this assay would be the PCR method of choice for the diagnosis of *S. lupi* in fecal samples.

## Conclusions

This study describes novel and efficient molecular assays for the detection of *S. lupi* DNA in feces from dogs with oesophageal spirocercosis and the quantification of fecal egg loads. The ITS-1 HRM qPCR showed the best performance and was superior to fecal flotation and other PCR assays. These new molecular assays are potential tools for the evaluation of treatment success and prognosis of spirocercosis in conjunction with clinical evaluation and the employment of imaging techniques.

## Additional files

Additional file 1: Table S1. DNA controls of dog parasites used in the study to test the specificity of the HRM qPCRs targeting different loci of *S. lupi*. (DOCX 12 kb)

Additional file 2: Figure S1. Limit of detection of the *cox*1 semi-nested PCR for *S. lupi*. DNA-standards with known epg concentration were run by triplicates, and the detection limit was estimated as the last concentration of which more than 67% of the samples were still positive. (PNG 867 kb)

Additional file 3: Figure S2. Correlation in the eggs per gram quantified in the HRM qPCRs for the ITS1 and 18S of *S. lupi* in fecal samples from dogs with spirocercosis. Logarithmic scales are used in both x- and y-axes. (PNG 16 kb)

## Abbreviations

18S: ribosomal subunit 18S
Cq: quantification cycle
*cytb*: cytochrome oxidase *b*
EPG: Eggs per gram
HRM: High-resolution melt
ITS1: Internal transcribed spacer 1
NTC: Non-template control
PCR: Polymerase chain reaction
qPCR: quantitative polymerase chain reaction

## References

[1] van der Merwe LL, Kirberger RM, Clift S, Williams M, Keller N, Naidoo V (2008). *Spirocerca lupi* infection in the dog: a review. Vet J.

[2] Mukaratirwa S, Singh VP. Prevalence of gastrointestinal parasites of stray dogs impounded by the Society for the Prevention of Cruelty to Animals (SPCA), Durban and Coast, South Africa. J S Afr Vet Assoc. 2010;81:123–5. 10.4102/jsava.v81i2.124 21247022

[3] Gholi-Toluei M, Amniattalab A, Rasouli S (2015). Oesophageal spirocercosis in stray dogs of Urmia. Res Op An Vet Sci.

[4] Aroch I, Markovics A, Mazaki-Tovi M, Kuzi S, Harrus S, Yas E, et al. Spirocercosis in dogs in Israel: a retrospective case-control study (2004–2009). Vet Parasitol. 2015;211:234–40. 10.1016/j.vetpar.2015.05.011 26012861

[5] Traversa D, Avolio S, Modrý D, Otranto D, Iorio R, Aroch I (2008). Copromicroscopic and molecular assays for the detection of cancer-causing parasitic nematode *Spirocerca lupi*. Vet Parasitol.

[6] Aroch I, Rojas A, Slon P, Lavy E, Segev G, Baneth G (2015). Serological cross-reactivity of three commercial in-house immunoassays for detection of *Dirofilaria immitis* antigens with *Spirocerca lupi* in dogs with benign oesophageal spirocercosis. Vet Parasitol.

[7] Markovics A, Medinski B (1996). Improved diagnosis of low intensity *Spirocerca lupi* infection by the sugar flotation method. J Vet Diagn Investig.

[8] Coskun SZ (1995). Diagnosis of *Spirocerca lupi* by IFAT in naturally infected dogs. Türk Parazitol Derg.

[9] Lam WY, Yeung AC, Tang JW, Ip M, Chan EW, Hui M (2007). Rapid multiplex nested PCR for detection of respiratory viruses. J Clin Microbiol.

[10] Yamamoto Y (2002). PCR in diagnosis of infection: detection of bacteria in cerebrospinal fluids. Clin Diagn Lab Immunol.

[11] Gasser RB. Molecular tools - advances, opportunities and prospects. Vet Parasitol. 2006;136:69–89. 10.1016/j.vetpar.2005.12.002 16457951

[12] Rojas A, Freedberg N, Markovics A, Gottlieb Y, Baneth G (2017). Influence of physical and chemical factors on the embryonation, hatching and infectivity of *Spirocerca lupi*. Vet Parasitol.

[13] Vrain T, Wakarchuk D, Lévesque A, Hamilton R (1992). Intraspecific rDNA restriction fragment length polymorphism in the *Xiphinema americanum* group. Fund Appl Nematol.

[14] Cherry T, Szalanski AL, Todd TC, Powers TO. The internal transcribed spacer region of *Belonolaimus* (Nemata: Belonolaimidae). J Nematol. 1997;29:23–9. PMC2619755 19274130

[15] Ye J, Coulouris G, Zaretskaya I, Cutcutache I, Rozen S, Madden TL (2012). Primer-BLAST: a tool to design target-specific primers for polymerase chain reaction. BMC Bioinformatics.

[16] Liu GH, Wang Y, Song HQ, Li MW, Ai L, Yu XL (2013). Characterization of the complete mitochondrial genome of *Spirocerca lupi*: sequence, gene organization and phylogenetic implications. Parasit Vectors.

[17] Jacobs DE, Fisher MA. Recent developments in the chemotherapy of *Toxocara canis*infection in puppies and the prevention of toxocariasis. In: Lewis JW, Maizels RM, editors. *Toxocara* and toxocariasis: clinical, epidemiological and molecular perspectives. London: Institute of Biology; 1993. p. 111–6.

[18] Landis JR, Koch GG (1977). The measurement of observer agreement for categorical data. Biometrics.

[19] Verweij JJ, Stensvold CR (2014). Molecular testing for clinical diagnosis and epidemiological investigations of intestinal parasitic infections. Clin Microbiol Rev.

[20] Rojas A, Rojas D, Montenegro VM, Baneth G (2015). Detection of *Dirofilaria immitis* and other arthropod-borne filarioids by an HRM real-time qPCR, blood-concentrating techniques and a serological assay in dogs from Costa Rica. Parasit Vectors.

[21] Mazaki-Tovi M, Baneth G, Aroch I, Harrus S, Kass PH, Ben-Ari T (2002). Canine spirocercosis: clinical, diagnostic, pathologic, and epidemiologic characteristics. Vet Parasitol.

[22] Christie J, Schwan EV, Bodenstein LL, Sommerville JE, van der Merwe LL (2011). The sensitivity of direct faecal examination, direct faecal flotation, modified centrifugal faecal flotation and centrifugal sedimentation/flotation in the diagnosis of canine spirocercosis. J S Afr Vet Assoc.

[23] Lavy E, Aroch I, Bark H, Markovics A, Aizenberg I, Mazaki-Tovi M (2002). Evaluation of doramectin for the treatment of experimental canine spirocercosis. Vet Parasitol.

[24] Giannelli A, Baldassarre V, Ramos RA, Lia RP, Furlanello T, Trotta M (2014). *Spirocerca lupi* infection in a dog from southern Italy: an "old fashioned" disease?. Parasitol Res.

[25] Durant JF, Irenge LM, Fogt-Wyrwas R, Dumont C, Doucet JP, Mignon B (2012). Duplex quantitative real-time PCR assay for the detection and discrimination of the eggs of *Toxocara canis* and *Toxocara cati* (Nematoda, Ascaridoidea) in soil and fecal samples. Parasit Vectors.

