# Supplementary material for: Detection and quantification of Spirocerca lupi by HRM qPCR in fecal samples from dogs with spirocercosis
Source: Parasit Vectors. 2017 Sep 19;10:435. doi: 10.1186/s13071-017-2374-3 (PMC5606040; doi:10.1186/s13071-017-2374-3)
Supplement: Supplementary file 1 — DNA controls of dog parasites used in the study to test the specificity of the HRM qPCRs targeting different loci of S. lupi. (DOCX 12 kb) [file 13071_2017_2374_MOESM1_ESM.docx]

**Table 1** Characteristics of the HRM qPCR assay performances

| Parameter | PCR assay | | |
| --- | --- | --- | --- |
|  | HRM qPCR for ITS1 | HRM qPCR for 18S | HRM qPCR for cytb |
| Efficiency (%) | 90 | 103 | 134 |
| Slope | -3.61 | -3.23 | -2.70 |
| y-intercept | 35.15 | 34.88 | 40.12 |
| *R*^2^-value | 0.9976 | 0.9962 | 0.9896 |
