# Supplementary figures and images for: Detection and quantification of Spirocerca lupi by HRM qPCR in fecal samples from dogs with spirocercosis
Source: Parasit Vectors. 2017 Sep 19;10:435. doi: 10.1186/s13071-017-2374-3 (PMC5606040; doi:10.1186/s13071-017-2374-3)

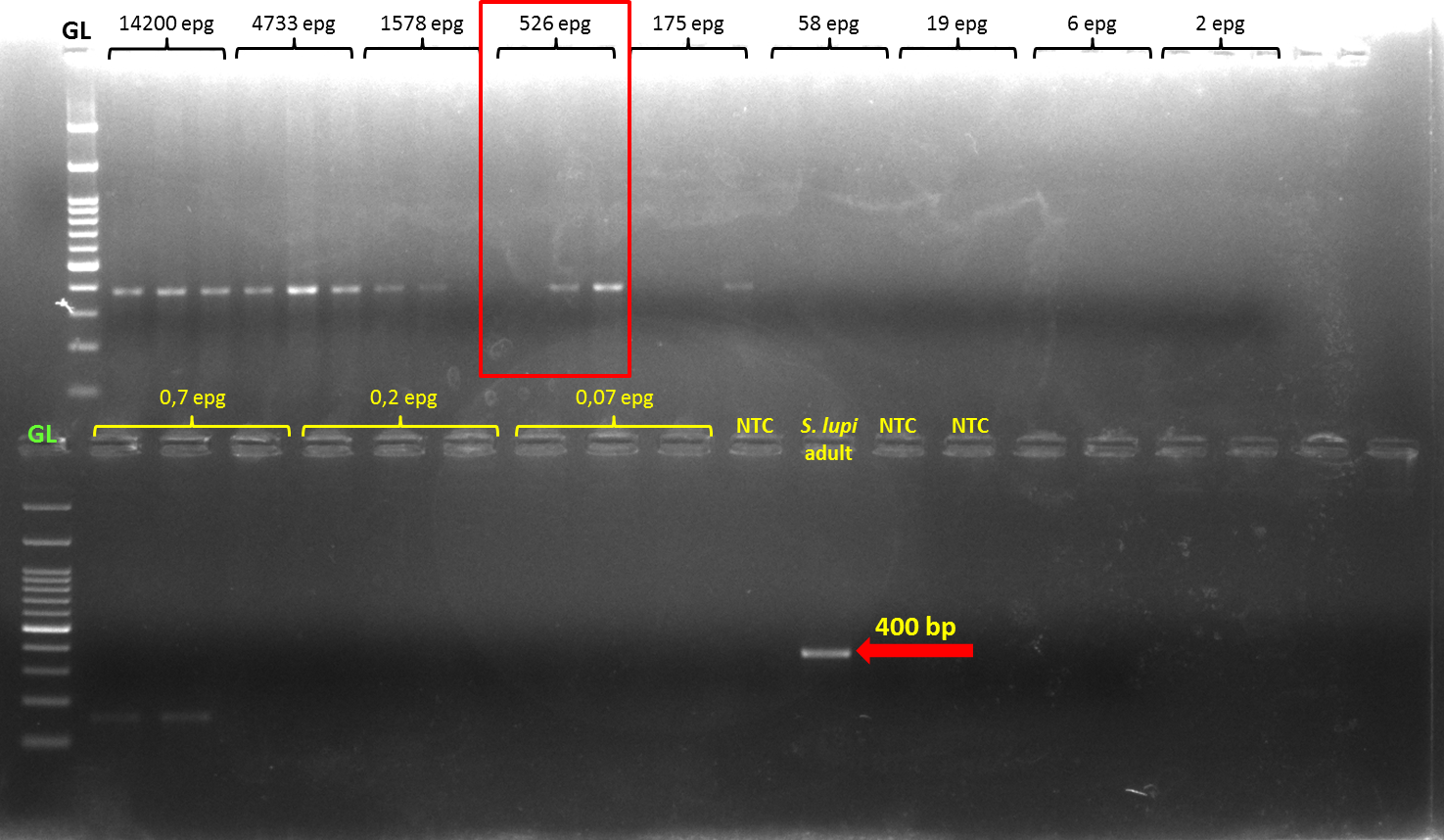

Supplement: Supplementary file 2 — Limit of detection of the cox1 semi-nested PCR for S. lupi. DNA-standards with known epg concentration were run by triplicates, and the detection limit was estimated as the last concentration of which more than 67% of the samples were still positive. (PNG 867 kb) [file 13071_2017_2374_MOESM2_ESM.png]

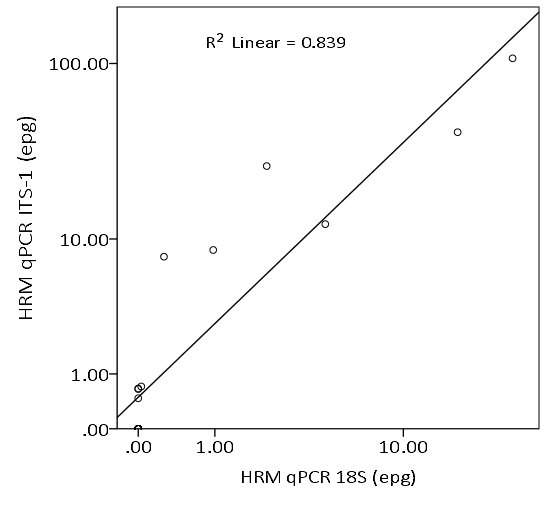

Supplement: Supplementary file 3 — Correlation in the eggs per gram quantified in the HRM qPCRs for the ITS1 and 18S of S. lupi in fecal samples from dogs with spirocercosis. Logarithmic scales are used in both x- and y-axes. (PNG 16 kb) [file 13071_2017_2374_MOESM3_ESM.png]
